# COVID-19 among patients with orthopedic surgery: our experience from the Middle East

**DOI:** 10.1186/s13018-021-02483-6

**Published:** 2021-05-25

**Authors:** Abolfazl Bagherifard, Peyman Arasteh, Mostafa Salehpour, Hooman Shariat Zadeh, Farid Najd Mazhar, Hasan Ghandhari, Mohammad Reza Bahaeddini, Pouria Tabrizian, Alireza Askari

**Affiliations:** 1 Bone and Joint Reconstruction Research Center, Shafa Orthopedic Hospital, Iran University of Medical Sciences, Tehran, Iran; 2 Shiraz University of Medical Sciences, Shiraz, Iran

**Keywords:** COVID-19, Fracture, Orthopedics, Hospital, Pandemic

## Abstract

**Background:**

We report our experiences with COVID-19 in one of the largest referral orthopedic centers in the Middle East and aimed to describe the epidemiology and clinical characteristics of these patients.

**Methods:**

During February 20 and April 20, 2020, patients who underwent orthopedic surgery and healthcare staff who were in contact with these patients were screened for COVID-19. To identify patients who were in the incubation period of COVID-19 during their hospital stay, all patients were tested again for COVID-19 4 weeks after discharge.

**Results:**

Overall, 1244 patients underwent orthopedic surgery (1123 emergency and 121 elective) during the study period. Overall, 17 patients were diagnosed with COVID-19 during hospital admission and seven after discharge. Among the total 24 patients with COVID-19, 15 were (62.5%) males with a mean (SD) age of 47.0±1.6 years old. Emergency surgeries were performed in 20 (83.3%) patients, and elective surgery was done in the remaining 4 patients which included one case of posterior spinal fusion, spondylolisthesis, acromioclavicular joint dislocation, and one case of leg necrosis. A considerable number of infections occurred in patients with intertrochanteric fractures (n=7, 29.2%), followed by pelvic fractures (n=2, 8.3%), humerus fractures (n=2, 8.3%), and tibial plateau fractures (n=2, 8.3%). Fever (n=11, 45.8%) and cough (n=10, 37.5%) were the most common symptoms among patients. Laboratory examinations showed leukopenia in 2 patients (8.3%) and lymphopenia in 4 (16.7%) patients. One patient with a history of cancer died 2 weeks after discharge due to myocardial infarction.

Among hospital staff, 26 individuals contracted COVID-19 during the study period, which included 13 (50%) males. Physicians were the most commonly infected group (n = 11), followed by operation room technicians (n = 5), nurses (n = 4), and paramedics (n = 4).

**Conclusions:**

Patients who undergo surgical treatment for orthopedic problems, particularly lower limb fractures with limited ambulation, are at a higher risk of acquiring COVID-19 infections, although they may not be at higher risks for death compared to the general population. Orthopedic surgeons in particular and other hospital staff who are in close contact with these patients must be adequately trained and given appropriate personal protective equipment during the COVID-19 outbreak.

## Introduction

Coronavirus disease 2019 or COVID-19 has now spread to include more than 200 countries worldwide [1].

The recent pandemic has put a large burden on global health and economy, and researches and institutions are constantly attempting to further understand the behavior of the disease in different settings. It is well-known that transmission of COVID-19 is facilitated in places such as hospitals where individuals are usually in close contact [2]. In the settings of orthopedic hospitals, transmission of COVID-19 is more concerning, as patients with fractures, particularly those with lower-limb fractures and limited ambulation, are highly susceptible to pulmonary infections [3]. Despite the importance of COVID-19 control in fracture patients, limited information is available regarding the outbreak of COVID-19 in orthopedic health care centers.

In this study, we report our experiences with COVID-19 among hospital staff and patients who underwent surgical treatment during the disease outbreak in the largest referral orthopedic center in Iran.

## Methods

### Study settings and design

This study was conducted in Shafa Hospital affiliated to Iran University of Medical Sciences, Tehran, Iran. The hospital is the oldest orthopedic hospital in Iran and is among the main referral centers for orthopedic surgery in the region. During February 20 and April 20, 2020, patients who underwent elective or emergency surgery in our orthopedic hospital as well as the healthcare staff who were in contact with these patients were screened for COVID-19. The screening criteria included a body temperature above 38°C; respiratory symptoms such as coughing, sputum production, sore throat, expectoration, rhinorrhea, hemoptysis, and dyspnea; gastrointestinal symptoms such as diarrhea, nausea or vomiting, and abdominal pain; and other symptoms including fatigue, anorexia, myalgia, headache, and dizziness. If individuals had any of the mentioned symptoms, a chest computed tomography (CT) scan was first obtained. In cases that had a suspicious CT scan for COVID-19, a polymerase chain reaction (PCR) test was requested. In cases of ground-glass opacity in CT scan or a positive PCR result, the patient was considered positive for COVID-19 and was treated accordingly. Aside from appropriate hospital care, specific treatment for COVID-19 at the time of study included hydroxychloroquine (200 mg per day) for 5 days. Other treatments were started case specific and according to the consultation of an infectious specialist. Patients who had a fever above 38°C along with dyspnea, as well as patients with typical COVID-19 symptoms such as anosmia and coughing, were also treated for COVID-19, despite a negative PCR test or CT scan for COVID-19. After the initial 5-day treatment course, a second chest CT scan was taken from each patients. In cases where a patient had persistent COVID-19 symptoms or ground-glass opacity was still present in the CT scan of the patients, another 5-day course of hydroxychloroquine was started. This process was repeated until the patient’s clinical symptoms and radiologic signs were resolved. Blood tests including white blood cell count, especially for evaluation of leukopenia and lymphopenia, were done for each patient regularly.

To identify patients who were in the incubation period of COVID-19 during their hospital stay, all patients were screened and tested again for COVID-19 4 weeks after discharge. Patients who were not available for post-discharge investigation for any reason were excluded from the study.

### Policies for orthopedic surgery during the COVID-19 pandemic

As little information existed on COVID-19, in our center similar to other centers in the country, most elective surgeries continued as routine up to 2 weeks after the announcement of the first cases of COVID-19 in Iran on February 19, 2021. After which, according to the instruction of the Ministry of Health of Iran, all elective surgeries were stopped, and only emergency surgeries were performed. Specifics on the changes in orthopedic practice in Iran have been described elsewhere [4].

As a result of these changes, number of orthopedic surgeries in our center decreased dramatically during the outbreak. After April 20, most elective orthopedic surgeries were restarted in our center, and number of elective surgeries increased similar to before the pandemic.

All patients were required to wear masks during their hospital admission and were isolated from other patients to prevent spread of infection. All surgeons and all health care workers were obligated to wear masks (normal surgical masks were available in most instances). Face shield was not generally used during the study period by health care workers.

### Ethical consideration

The study protocol was approved by the Institutional Review Board of Iran University of Medical Sciences. All participants gave their written and informed consent for their data to be used for research purposes; moreover, no patient personnel data was released.

### Statistical analysis

Data was managed using the Statistical Package for Social Sciences (SPSS Inc., Chicago, IL, USA) for windows, version 25. Quantitative variables with normal distribution are reported as means and standard deviations (SD), and quantitative variables without a normal distribution are reported as medians and interquartile range (IQR). Descriptive data are shown as frequency and percentage.

## Results

### COVID-19 among patients

In total, 1244 patients underwent surgical treatment for orthopedic problems (1123 emergency and 121 elective surgeries) in our center during the study period. Overall, 17 patients were diagnosed with COVID-19 during hospital admission. The rest of the patients (n = 1227) were screened within 4 weeks after discharge. Two hundred and thirty-seven patients (19.3%) were not available for follow-ups and were excluded from the study. COVID-19 was investigated in the remaining 1007 patients, among which seven patients had clinical symptoms of COVID-19 and were referred for complementary COVID-19 testing.

Among the total 24 patients with COVID-19, 15 were (62.5%) males and 9 (37.5%) were females with a mean (SD) age of 47.0 ± 1.6 years old (ranging from 17 to 83 years old). Emergency surgeries were performed in 20 (83.3%) patients. A considerable number of infections occurred in patients with intertrochanteric fractures (29.2%), followed by pelvic fractures (8.3%), humerus fractures (8.3%), and tibial plateau fractures (8.3%). Majority of patients (70.8%) were Iranian. Median (IQR) hospital stay was 4 [3, 5] days (range from 1 to 31 days). Fever (45.8%) and cough (37.5%) were the most common symptoms among patients. Laboratory examinations showed leukopenia in 2 patients (8.3%) and lymphopenia in 4 (16.7%) patients. The mean (SD) duration of treatment for COVID-19 with hydroxychloroquine was 5.83 ± 1.90 days (ranging from 5 to 10 days). Characteristics of patients are shown in Table 1.

**Table 1:** Characteristics of patients undergoing orthopedic surgery with COVID-19

Variables	Statistics
Age, yrs	47.0 ± 1.6
Sex, no. (%)	
Male	15 (62.5)
Female	9 (37.5)
Nationality, no. (%)	
Iranian	17 (70.8)
Non-Iranian	7 (29.2)
Underlying disease, no. (%)	
None	17 (70.8)
Diabetes	3 (12.5)
Asthma	1 (4.2)
Cardiac	1 (4.2)
Hypertension, cardiac disease, and lung disease	1 (4.2)
Hypertension and diabetes	1 (4.2)
CT scan in favor of COVID-19	
Yes	22 (91.7)
No	2 (8.3)
Symptoms, no. (%)	
Fever	11 (45.8)
Headache	2 (8.3)
Cough	9 (37.5)
Myalgia	3 (12.3)
Dyspnea	4 (16.7)
Anosmia	2 (8.3)
Respiratory distress	6 (25)
Gastrointestinal symptoms	2 (8.3)
Hospital stay, days	5.58 ± 5.97
Median (IQR)	4 (3, 6)
Surgery type, no. (%)	
Emergency	20 (83.3)
Elective	4 (16.7)
Surgery, no. (%)	
Intertrochanteric fracture	7 (29.2)
Pelvic fracture	2 (8.3)
Humerus fracture	2 (8.3)
Tibial plateau fracture	2 (8.3)
Acromioclavicular joint dislocation	1 (4.2)
Acetabular fracture	1 (4.2)
Bimalleolar fracture	1 (4.2)
Both bone fracture	1 (4.2)
Distal femoral fracture	1 (4.2)
Leg necrosis	1 (4.2)
Patellar fracture	1 (4.2)
Posterior cruciate ligament avulsion	1 (4.2)
Posterior spinal fusion	1 (4.2)
Shoulder dislocation	1 (4.2)
Spondylolisthesis	1 (4.2)
Smoker, no. (%)	
Yes	4 (16.7)
No	20 (83.3)
Drug abuse, no. (%)	
Yes	4 (16.7)
No	20 (83.3)
Duration of treatment, days	5.83 ± 1.90

All plus-minus values are means and standard deviations

One patient who had a history of cancer died 2 weeks after discharge due to myocardial infarction. COVID-19 resolved in the rest of the patients.

### COVID-19 in hospital staff

The hospital currently has 470 staff members including 400 hospital workers, 20 faculty members, and 50 residents and fellows. In total, 26 individuals contracted COVID-19 during the study period, which included 13 (50%) males and 13 (50%) females with a mean (SD) age of 34.0 ± 6.7 years old (ranging from 24 to 52 years old). Physicians were the most commonly infected group (n = 11) among the hospital staff (four orthopedic surgeons and seven orthopedic residents) followed by operation room technicians (n = 5), nurses (n = 4), paramedics (n = 4), a security guard (n = 1), an occupational therapist (n = 1), and an employee from the hospital administrative affairs (n = 1). Fever (42.3%), cough (38.5%), and myalgia (23.1%) were the most common symptoms among hospital staff. None of the infected staff had a recent history of traveling abroad. Leukopenia was seen in two patients (7.7%); moreover, 4 patients had lymphocytopenia (15.4%). The mean (SD) treatment duration was 5.6 ± 1.7 days (ranging from 0 to 10 days). All hospital staff successfully recovered from COVID-19, and no death was recorded in this series. The clinical characteristics of hospital staff who contracted COVID-19 are shown in Table 2.

**Table 2:** Characteristics of hospital staff with COVID-19

Variables	Statistics
Age, yrs	34.0 ± 6.8
Sex, no. (%)	
Male	`13 (50)
Female	13 (50)
Underlying disease, no. (%)	
None	25 (96.2)
Diabetes	1 (3.8)
Asthma	0
Cardiac	0
Symptoms, no. (%)	
Fever	11 (42.3)
Headache	5 (19.2)
Cough	10 (38.5)
Myalgia	6 (23.1)
Dyspnea	4 (15.4)
Anosmia	3 (11.5)
Respiratory distress	1 (3.8)
Gastrointestinal symptoms	2 (7.7)
Hypertension, cardiac disease, and lung disease	0
Hypertension and diabetes	0
Smoker, no. (%)	
Yes	3 (11.5)
No	23 (88.5)
Drug abuse, no. (%)	
Yes	0
No	26 (100)
Duration of treatment, days	5.65 ± 1.72

All plus-minus values are means and standard deviations

## Discussion

We described our experiences in the largest orthopedic referral center in Iran during the COVID-19 outbreak. Overall, 1.7% of patients who were referred to our center for emergency surgeries and 3.3% of patient who referred to our center for elective surgeries were diagnosed with COVID-19.

COVID-19 has affected orthopedics in different aspects [5–8]. On the other hand, reports on orthopedic surgery during the COVID-19 outbreak have been scarce. Mi et. al. reported the characteristics of 10 fracture patients with COVID-19 in Wuhan, China [9]. In their series, intertrochanteric fractures were the most frequent type of fractures (50%). Three patients in their center underwent surgery, while other patients were managed conservatively. The most common symptoms of their patients were fever, cough, and fatigue, which were seen in 70% of their patients, followed by dyspnea (50%) and sore throat (40%). Headache, dizziness, abdominal pain, and vomiting were less frequent symptoms in their series. Four deaths (40%) occurred among their patients. Accordingly, they concluded that early prognosis of COVID-19 in patients with fractures tends to be poor compared to that reported for adult patients without fractures. They suggested non-operative treatment, particularly for older individuals during the outbreak. Similar to the report from China, intertrochanteric fractures were the most frequent orthopedic problem among our patients (29.2% vs. 50%). Fever (45.8%) and cough (37.5%) were the most common symptoms of COVID-19 in our report as well. Unlike the aforementioned report, dyspnea was only reported in 16.7% (vs. 50%) of our patients. Furthermore, the rate of lymphopenia was significantly higher in their report compared to ours (60% vs. 7.1%).

In our study, only one patient died (4%) after acquiring COVID-19, although the cause of death seemed unrelated to COVID-19. This was incoherent with the report from Wuhan, China, and does not support the notion that individuals with fractures are at higher risks of death due to COVID-19. From another perspective, up to June 15, 2020, in Iran, a total of 187,427 have been diagnosed with COVID-19 and 8837 have died due to the disease. This shows that death rates reported among our fracture patients who acquired COVID-19 is very similar to that of the normal population (4.7% vs. 4% in our report).

Another study from New York, USA [10], reported on ten hip fracture patients who acquired COVID-19. All their patients were older than 60 years old, and only two of their patients became symptomatic. In their report, only one patient had an abnormal radiography. Furthermore, they reported that only one of their patients had a re-admission for COVID-19. Although in this study no mortality was recorded among patients with hip fracture surgery, other reports from Spain and Italy [11–13] have reported higher mortality rates among patients with hip fractures who underwent surgery during the COVID-19 pandemic. This may be due to the fact that patients with hip fractures are usually older and have more comorbidities which render them susceptible to severe symptoms from COVID-19.

Up to June 15, 2020, in Iran that has a population of 83,950,295 (https://www.worldometers.info/world-population/iran-population/), a total of 187,427 individuals have acquired COVID-19; this makes an infection rate of 0.22%. In our report among a total of 1244 who underwent orthopedic surgery during the study period, 24 patients (1.9%) acquired COVID-19. The high infection rate among patients who undergo surgical treatment for orthopedic problems could be attributed to multiple factors. First is that fractures are associated with stress which results in the expression of a variety of inflammatory mediators, thereby suppressing the patients’ immunity and increasing the likelihood of respiratory infections [14, 15]. At the same time, the majority of patients with hip fractures are old and already have many underlying conditions compromising their immune and physical condition [16, 17]. In addition, patients with limited ambulation are at higher risks of death after surgery [18]. Therefore, much care is needed to prevent COVID-19 infection in these patients. Suspending or minimizing elective surgeries could be one of the best choices to reduce the risk of COVID-19 infection during the pandemic, as done in several centers in the world [19, 20].

Among a total of 470 plus hospital staff members during our study period, 26 (5.5%) contracted the novel virus. Orthopedic surgeons who are dealing with fracture patients during the COVID-19 pandemic are especially at greater risks of infection [21]. In the present series, surgeons were the most commonly infected hospital staff members (42.3%). Other groups including operation room technicians and nurses are also at high risks for acquiring COVID-19. Considering that many centers have recovered their number of elective surgeries [22], immediate measures are needed to address the challenges orthopedic surgeons are facing during the pandemic [23].

The present study was not without limitations. We did not exclude patients who were already infected with COVID-19 infection at the time of referral for surgery; moreover, we included patients who showed symptoms up to 4 weeks after their hospital discharge, as a result patients who may have contracted the virus before and after their hospital admission may also have entered the study. In addition, as we initially screened patients based on clinical symptoms and not by PCR or CT scan, many patients that were asymptomatic may have been missed in this study. Our treatment protocol included the use of hydroxychloroquine which is currently not recommended for treatment of COVID-19-related infection, and this was mainly due to the fact that our study was conducted during the early phases of the outbreak, during which little evidence existed on the treatment of COVID-19. A relatively high volume of patients (19.3%) were lost to follow-up and excluded from study which may have affected our results.

## Conclusions

Patients who undergo surgical treatment for orthopedic problems, particularly lower limb fractures with limited ambulation, are at a higher risk of acquiring COVID-19 infections, although they may not be at higher risks for death compared to the general population. Orthopedic surgeons in particular and other hospital staffs who are in close contact with these patients must be adequately trained and given appropriate personal protective equipment during the COVID-19 outbreak.

## Data Availability

All authors and institutions can request data related to the study from the corresponding author.
